# Tailored Rehabilitation Protocol on Hypoxic-Ischemic Encephalopathy: A Case Report

**DOI:** 10.7759/cureus.69234

**Published:** 2024-09-11

**Authors:** Disha S Songire, H. V. Sharath

**Affiliations:** 1 Department of Neurophysiotherapy, Ravi Nair Physiotherapy College, Datta Meghe Institute of Higher Education and Research, Wardha, IND; 2 Department of Pediatric Physiotherapy, Ravi Nair Physiotherapy College, Datta Meghe Institute of Higher Education and Research, Wardha, IND

**Keywords:** hypo-ischemic encephalopathy, neonates, oromotor stimulation, physical therapy, rehabilitation

## Abstract

Hypoxic-ischemic encephalopathy (HIE) is a dangerous illness that happens when a newborn's brain does not receive adequate oxygen and blood flow, which is frequently caused by difficulties during pregnancy or delivery. This case focuses on the many outcomes and treatment techniques for HIE, which can result in serious effects such as cerebral palsy and epilepsy. The hypoxic-ischemic event can occur before, during, or after birth, and it is probable causes include maternal variables, delivery procedures, placental difficulties, or infant disorders. In this case report, a male infant diagnosed with HIE required ventilator support and subsequently developed pneumonia. He experienced respiratory problems and feeding challenges. The report illustrates that treating neonatal HIE with a combination of chest physiotherapy and oromotor stimulation (OMS) may have beneficial effects. Improvements in respiratory and neurodevelopmental functioning were noted, highlighting the therapy's potential to aid recovery and enhance overall well-being.

## Introduction

Hypoxic-ischemic encephalopathy (HIE) accounts for a significant percentage of encephalopathic neonates. Despite advances in perinatal care, moderate-to-severe acute perinatal HIE in late preterm and term newborns is still a leading cause of mortality, immediate brain damage, and long-term neurodevelopmental disability. Neonatal encephalopathy is a clinical illness of impaired brain function that appears early in life, with a prevalence of 1/1000 to 6/1000 live births. Impaired cerebral blood flow in the presence of hypoxia is the primary cause of brain injury after intrapartum hypoxia-ischemia [1].

An important contributing factor to cerebral palsy and related developmental disorders in children is hypoxia-ischemia during the prenatal stage. Because of its high prevalence (2/1000 births) and lifetime persistence, cerebral palsy is one of the most expensive neurologic disabilities. The most frequent cause of hypoxic injury in term infants is intrauterine asphyxia resulting from circulation issues like placental abruption, clotting of the placental arteries, or inflammatory processes [2]. Placental abruption, rupture of the uterus, embolism of amniotic fluid, tight nuchal cord, cord prolapse/avulsion, hemorrhage in the mother, trauma or cardiac, respiratory arrest, severe and prolonged fetal bradycardia, and extended labor are among these events [3].

The cerebral arteries use a survival mechanism to reroute blood flow from the anterior circulation to the posterior circulation when there is a slight drop in blood supply to the fetal brain. This preserves critical brain regions for immediate survival. However, the basal ganglia and thalami may also sustain injury in situations involving acute hypoxia and a significant decrease in cerebral blood flow. A series of actions are set off by the decreased cerebral perfusion, beginning with an energy crisis referred to as "primary energy failure" [4]. This emergency catalyzes the ischemia cascade, a series of harmful occurrences that follow the original injury. The combination of oxidative stress, excitotoxicity, intracellular calcium buildup, mitochondrial malfunction, and inflammation interacts intricately with the previous processes to increase the overall amount of brain damage. Three unique phases characterize the emergence of HIE: primary neuronal death in the immediate phase, latent phase, and secondary energy failure/delayed neuronal death in the final phase [5,6].

Managing respiratory issues in neonates with HIE is one of the major physiotherapy challenges. Enhancing lung function and efficiently eliminating pulmonary secretions is the goal of chest physical therapy (CPT). Vibration, percussion, and postural drainage are all useful methods for releasing secretions and preventing respiratory infections. These actions may improve the infants' overall respiratory health by lowering the risk of pneumonia and other respiratory illnesses, according to research [7]. It is understood that safe and efficient nutritive sucking (NS) involves the synchronized actions of sucking, swallowing, breathing, and esophageal function. All of these activities in the "nutritive sucking route" work together to convey a milk bolus from the mouth cavity to the stomach quickly and safely [8]. 

Infants with HIE usually have feeding problems as a result of decreased oromotor function. Physiotherapy, particularly oromotor stimulation (OMS), improves feeding efficiency and increases coordination of sucking, swallowing, and breathing. OMS is the manipulation of the lips, jaw, tongue, and soft palate prior to feeding, with or without NS or non-nutritive sucking (NNS) events, in order to improve preterm infant sucking and feeding capacities. Research indicates that feeding preterm infants can result in faster weight gain and shorter transition times. Oromotor exercises that involve tactile stimulation of the tongue, cheeks, and lips have the potential to significantly increase feeding capacity while reducing the need for other feeding methods [9-13].

## Case presentation

A 2.7 kg male neonate was born to gravida 1, para 1, living 1 (G2P1L1), mother via lower (uterine) segment cesarean section input/view/output (LSCS I/v/o) breech presentation on 04/05/2024; the baby did not cry immediately after birth and was referred to the hospital for further management. The child has multiple episodes of seizures, and further e/o of convulsions with altered aspirate was observed. Therefore, routine blood investigations were conducted, revealing the following results from the complete blood count: hemoglobin (Hb): 18.6, total leukocyte count (TLC): 12,700, platelet count: 0.9, hematocrit (HCT): 59.7; coagulation profile: prothrombin time (PT): 27.9, international normalized ratio (INR): 2.46, and C-reactive protein (CRP): 0.400, as detailed in Table 1. Then, the baby was intubated and maintained saturation on a ventilator in packed cell volume (PCV) mode with FiO_2 _at 40%, PEEP at 5 cm H_2_O, and was placed in a Mira cradle for 72 hours. The baby did not maintain target saturation, so a second echo was done, which showed severe pulmonary hypertension (PAH). Chest X-ray was done, and endotracheal (ET) culture showed growth of gram-negative bacilli ventilator-associated pneumonia was developed, so the baby was referred for physiotherapy for further management. The baby improved clinically and was maintaining saturation, so it was planned for extubation.

**Table 1:** Routine blood investigation CBC: complete blood count; Hb: hemoglobin; TLC: total leukocyte count; HCT: hematocrit; PT: prothrombin time; INR: international normalized ratio; CRP: C-reactive protein

CBC		
Test	Result	Normal range
Hb	18.6 g/dL	13.5-17.5 g/dL (male), 12.0-15.5 g/dL (female)
TLC	12700/µL	4,000-11,000/µL
Platelet count	0.9x10^5^/µL	1.5-4.5x10^5^/µL
HCT	59.70%	38.3%-48.6% (male), 35.5%-44.9% (female)
PT	27.9 seconds	11-13.5 seconds
INR	2.46	0.8-1.2
CRP	0.400 mg/dL	<0.3 mg/dL

Prenatal history

A normotensive pregnancy ensued, with the mother attending prenatal consultations on a regular schedule. Her physiological status remained stable throughout the gestational period.

Natal history

An elective cesarean section was used to deliver a full-term male child in view of breech presentation. Prenatal ultrasounds revealed no abnormalities.

Postnatal history

The neonate baby did not cry immediately after birth, and the Apgar score was six at five minutes.

On examination

A baby with a head circumference of 33 cm, a length of 55 cm, and a chest circumference of 36 cm. However, the baby showed signs of cyanosis. Primitive reflexes play a significant role in neonatal neurological evaluation. Neuro-muscular evaluation in neonates is an important aspect of newborn assessment since it measures the baby's neurological and muscular development. Here is a general summary of what a healthcare provider could assess during a neuro-muscular evaluation in neonates, which is noted in Table 2.

**Table 2:** Primitive reflexes

Palmar reflex	Present
Plantar reflex	Present
Sucking reflex	Absent
Rooting reflex	Absent

Cardiopulmonary examination

The cardiorespiratory examination in newborns is a critical component of the newborn assessment, as it facilitates the early identification of potential issues within the circulatory and respiratory systems. Slightly rapid and shallow breathing patterns suggest that the neonate may be experiencing tachypnea (elevated respiratory rate). Additionally, shallow breaths could indicate a reduced tidal volume or diminished air intake per breath.

Symmetry of Chest

Bilaterally symmetrical chest expansion suggests that both sides of the chest expand equally during respiration, indicating that there is no asymmetry or obstruction in the lungs or airways.

Inspiratory-to-Expiratory Ratio

The inspiratory-to-expiratory (I:E) ratio of 1:2 indicates that the duration of the expiration phase is twice as long as that of the inspiration phase. 

Rehabilitation protocol

To prevent more difficulties, the neonatal care team realized how important it was to manage the neonate's feeding challenges and secretions. Chest physiotherapy was started to assist in clearing up the secretions and enhance lung function. Oral motor stimulation was also employed to improve the neonate's sucking and swallowing reflexes. Chest physiotherapy involved moderate vibrations and suctioning to clear secretions, whereas OMS featured gentle massage and stimulation of the neonate's mouth and tongue to improve sucking and swallowing ability, as mentioned in Table 3.

**Table 3:** Physiotherapy protocol administered to the patient NNS: non-nutritive sucking, KMC: kangaroo mother care

Treatment	Description	Intensity
Percussion	Gently percussing the chest and back with cupped hands or a soft percussor to loosen secretions	30 percussions for five sets
Vibrations	Gentle vibrations are applied to the chest wall during exhalation to move mucus toward the larger airways	30 vibrations for five sets
Thoracic squeeze technique	Sustained compression of the chest wall with the hand above the chest is applied	three compressions with a 5-sec hold, one set
Oral awareness	Gentle stroking of lips, cheeks, and around the mouth using a soft cloth or finger	10 repetitions, one set
Palatal stimulation	Applying light pressure on the hard palate using a finger	10 repetitions, one set
Lip closure support	Encouraging lip closure by applying slight pressure on the upper lip	10 repetitions, one set
Oral massage	Using circular motions inside the cheeks and along the gums with a finger	10 repetitions, one set
NNS	Offering a clean finger or pacifier for the neonate to suck on	10 repetitions, one set
Jaw stabilization	Providing gentle support to the jaw to assist with sucking movement	10 repetitions, one set
Tactile stimulation	Using different Textures (e.g., pacifier or finger) around the mouth to enhance sensory input	10 repetitions, one set
KMC	The infant's head is turned to the side to keep the airway clear, and their legs are flexed in a frog-like position, with a cloth or wrap used to secure the baby against the mother’s chest	1 hour

**Figure 1:**
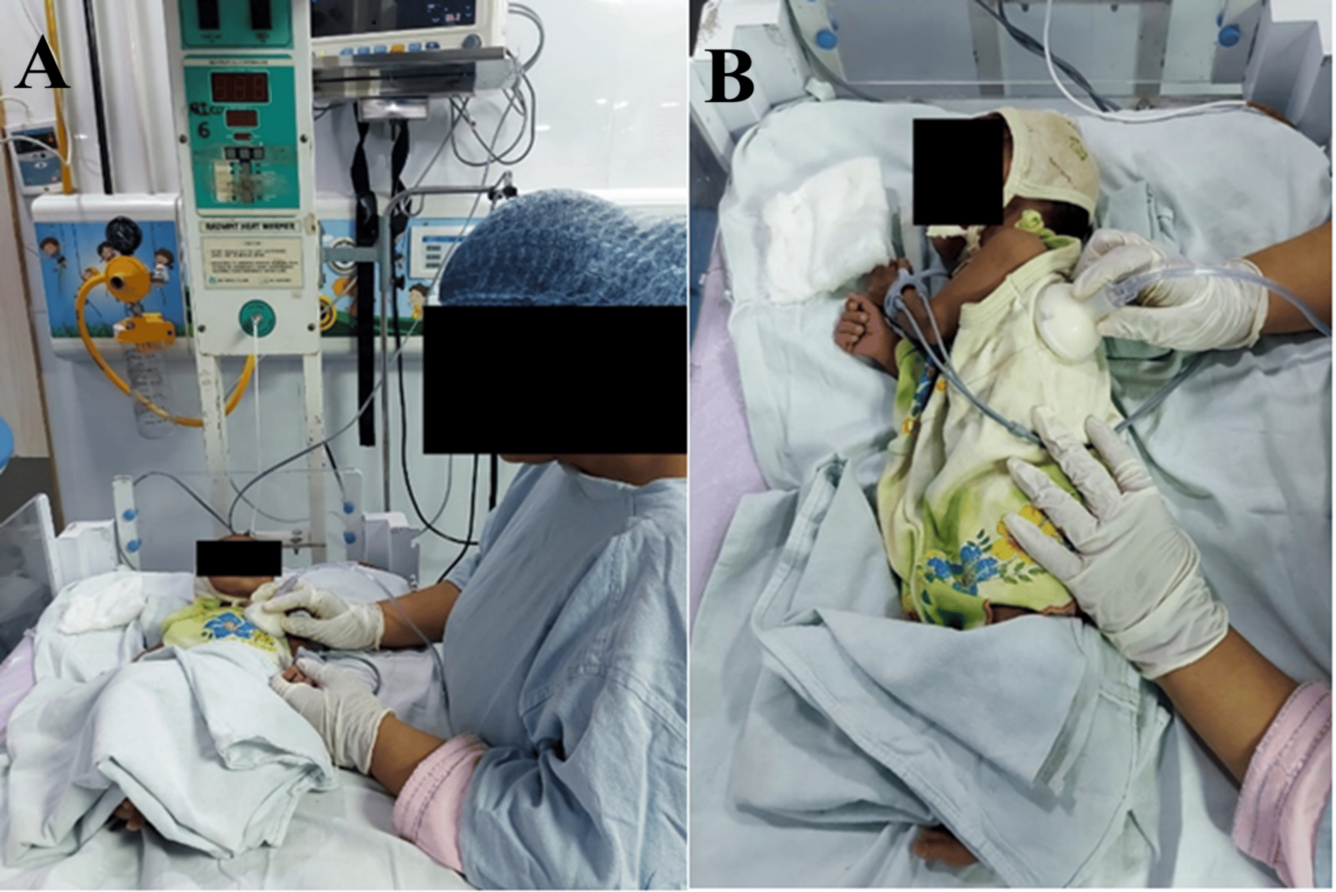
Mechanical chest vibrations: (A) in a supine-lying position and (B) in a side-lying position

Mechanical chest vibrations are a therapeutic technique used to help clear mucus from the lungs, and they can be performed in different positions to target specific areas. In the supine lying position, the patient lies on their back on a flat surface. The caregiver places their hands on the patient's chest, over the target area, usually the upper or middle lobes of the lungs. By rapidly contracting and relaxing the muscles in their arms and shoulders, the caregiver produces rhythmic vibrations in the chest. These vibrations are typically applied during the patient's exhalation phase for 3-5 minutes. The primary purpose of this technique is to loosen mucus in the lungs, making it easier for the patient to cough it up and clear their airways. In the side-lying position, the patient lies on their side with their bottom arm extended and the top arm resting comfortably, sometimes with a pillow placed between the knees for additional support. The caregiver places their hands on the side of the patient's chest, over the target area, which is often the lower lobes of the lungs. The same rhythmic vibration technique is used, applying pressure during the exhalation phase for 3-5 minutes. This position allows the caregiver to target different areas of the lungs, particularly beneficial for draining specific lobes that are more accessible in this position. Both techniques are essential components of chest physiotherapy, often used in combination with other methods such as postural drainage and percussion to improve lung function and overall respiratory health mentioned in Figure 1a, 1b.

Stroking

**Figure 2:**
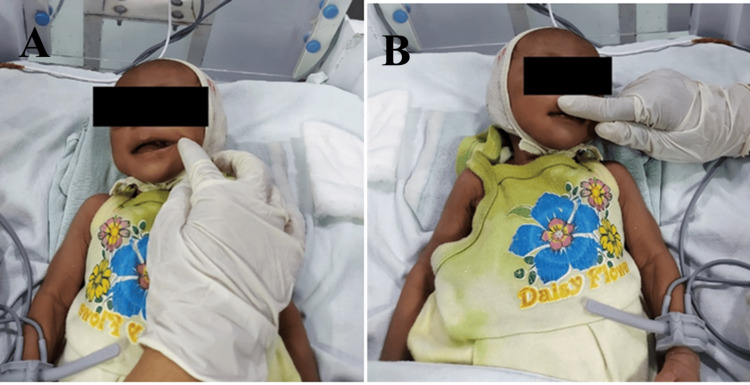
OMS: A) stroking and B) perioral pressure OMS: oromotor stimulation

Stroking is a gentle, repetitive motion applied to stimulate the muscles and nerves in the oral area. The caregiver uses their fingers or a soft tool to lightly stroke the inside and outside of the patient's cheeks, lips, and around the mouth. This technique helps increase sensory awareness, improve muscle tone, and promote coordinated movements necessary for effective feeding and speech. Stroking is typically done in a calm and rhythmic manner to avoid overstimulation and to encourage relaxation and comfort in the patient perioral pressure involves applying gentle, steady pressure around the mouth and lips. The caregiver uses their fingers to press and hold specific points on the face, such as the cheeks, chin, and above the upper lip. This technique helps to increase muscle tone and control, improve sensory feedback, and enhance oral motor skills. Perioral pressure is particularly beneficial for individuals with low muscle tone or those who have difficulty maintaining lip closure and controlling saliva. The application of pressure should be firm yet gentle, ensuring the patient remains comfortable throughout the process. Both stroking and perioral pressure are essential components of oromotor therapy, aimed at improving the functional abilities of individuals with oral motor challenges. These techniques are often incorporated into a comprehensive therapy program that may include other exercises and interventions to support feeding, speech, and overall oral health (Figure 2A, 2B).

Thoracic squeezing technique

**Figure 3:**
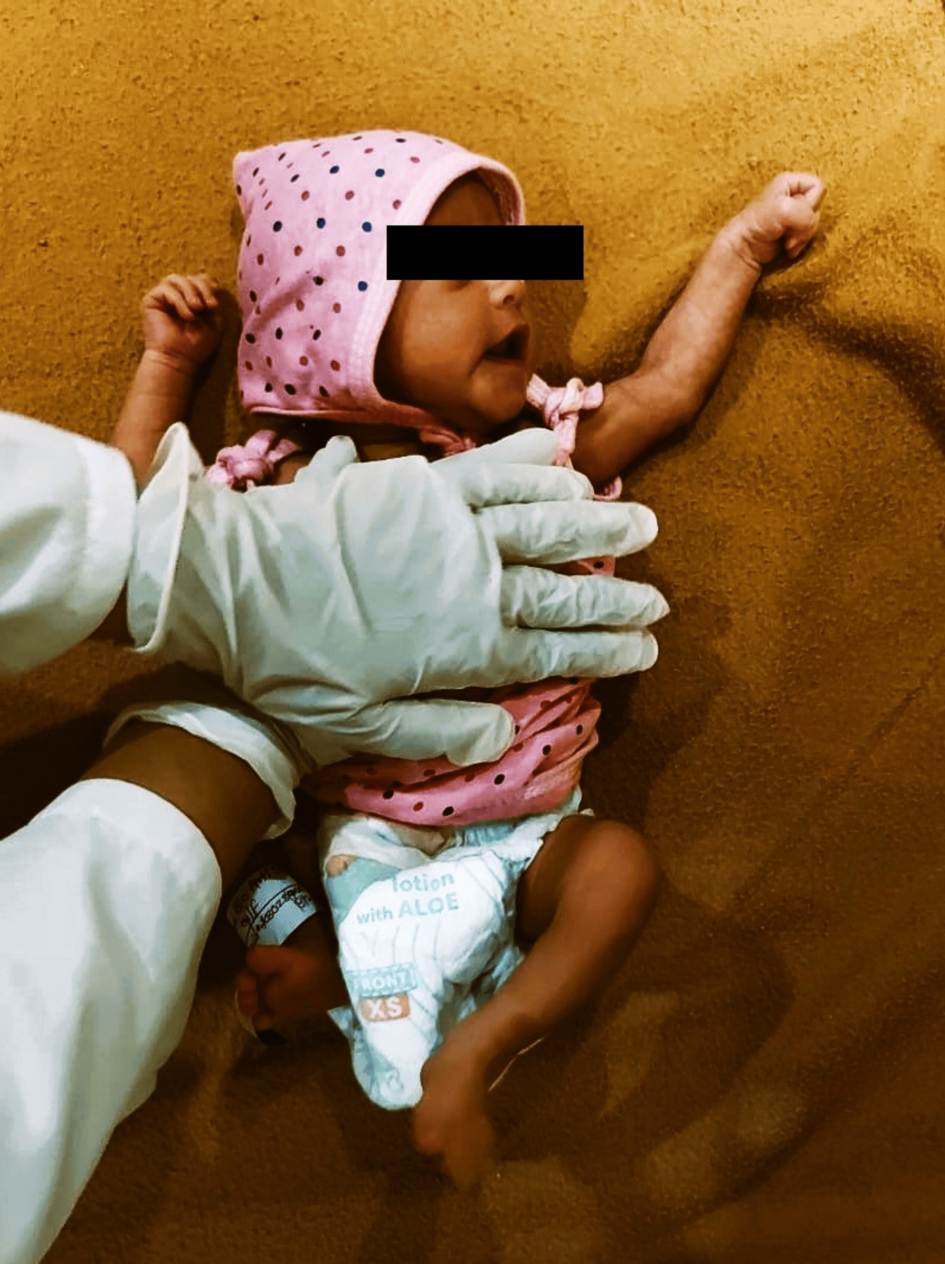
Application of TST TST: thoracic squeezing technique

It is a chest physiotherapy method designed to aid in the mobilization and clearance of lung secretions, thus improving respiratory function. To begin, the patient is positioned either lying on their back (supine position) or on their side (side-lying position) on a flat surface. The caregiver places their hands on both sides of the patient's ribcage, ensuring that the thumbs are positioned toward the front of the chest and the fingers are wrapped around the sides and back of the ribcage. During the patient’s exhalation phase, the caregiver applies gentle, yet firm, pressure to the ribcage, squeezing it inward and downward. This squeezing action is coordinated with the patient's breathing pattern to enhance the effectiveness of the technique. The pressure is maintained until the end of the exhalation, then gently released as the patient begins to inhale. This rhythmic squeezing motion helps to loosen and mobilize mucus, making it easier for the patient to cough it up and clear their airways. The duration of the thoracic squeezing typically lasts for several minutes, depending on the patient's tolerance and the specific therapeutic goals. The technique can be repeated multiple times during a therapy session, often in combination with other chest physiotherapy methods like postural drainage and percussion for optimal results (Figure 3).

Kangaroo mother care

**Figure 4:**
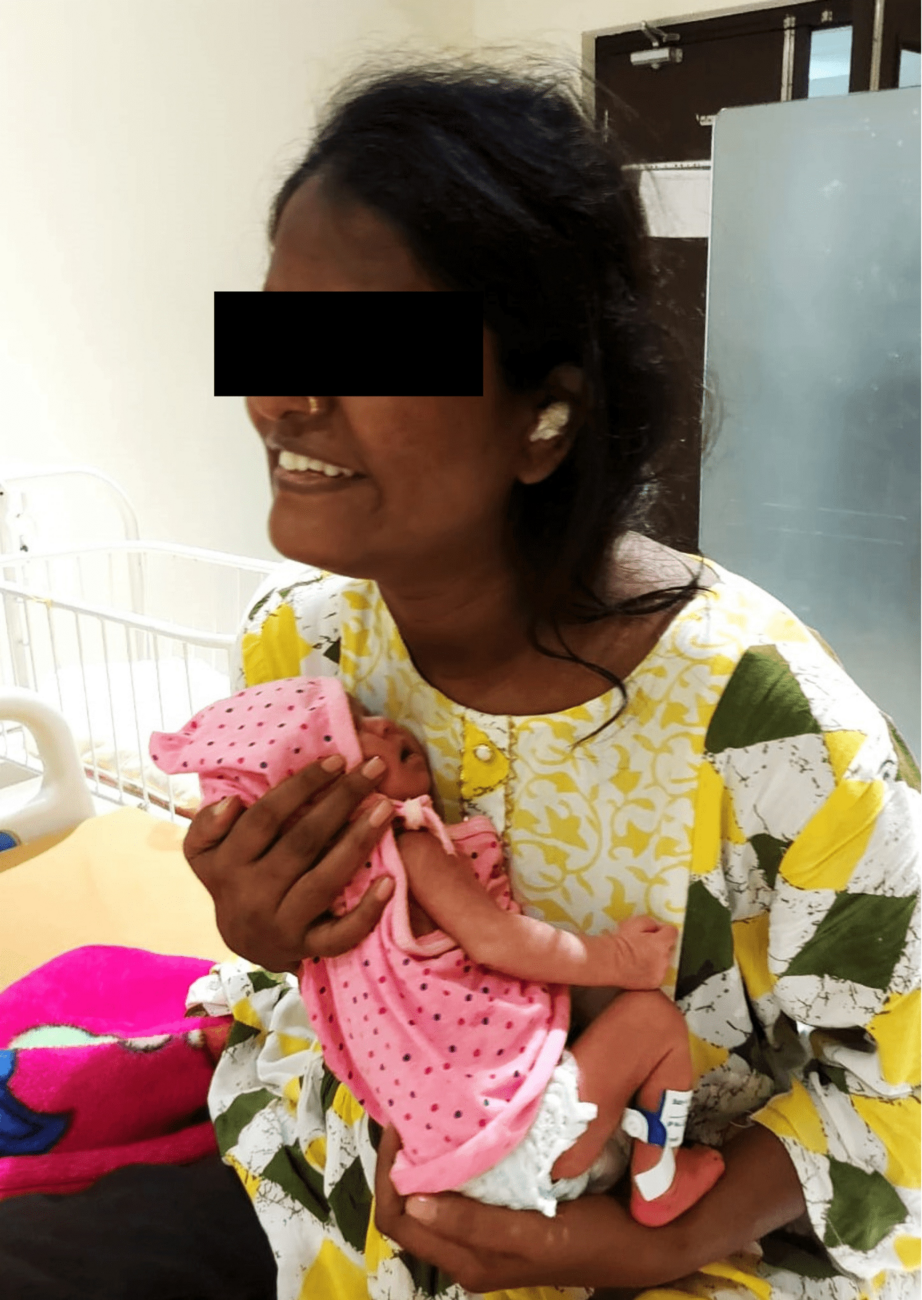
KMC KMC: kangaroo mother care

Kangaroo mother care (KMC) is a highly beneficial method of caring for preterm and low birth-weight infants that involves prolonged skin-to-skin contact between the mother and baby. In this technique, the infant is placed upright against the mother’s bare chest, ensuring direct skin-to-skin contact. This positioning helps maintain the infant’s body temperature more effectively than an incubator, promotes frequent and exclusive breastfeeding, and enhances the emotional bond between the mother and baby. The infant's head is turned to the side to keep the airway clear, and his legs are flexed in a frog-like position, with a cloth or wrap used to secure the baby against the mother’s chest. KMC has been shown to significantly reduce mortality rates among preterm and low birth-weight infants, support better weight gain, shorten hospital stays, and improve neurodevelopmental outcomes. For mothers, KMC fosters a sense of empowerment, reduces stress and anxiety, and enhances lactation by stimulating milk production. This method can be practiced in hospitals, neonatal intensive care units (NICUs), and at home, with education and support from healthcare providers to ensure mothers and families are comfortable and confident in its application. The involvement of fathers and other family members is also encouraged to support the mother and provide additional bonding opportunities. KMC is a simple, cost-effective, and highly effective practice that leverages the natural benefits of skin-to-skin contact to promote the health and well-being of both infants and mothers, making it a valuable approach in neonatal care (Figure 4).

Outcome measures that are taken pre- and post-intervention are mentioned in Table 4.

**Table 4:** Outcome measures

Outcomes measures	Pre-treatment	Post-treatment
Ballard score	16	18
Breastfeeding scale	6/10	8/10
Weight of the baby	2.7 kg	3.5 kg

## Discussion

HIE is a major disease in neonates that necessitates multidisciplinary management. This method requires both chest physiotherapy and OMS. In the case of infants with HIE, this is significant because they have reduced respiratory function, which usually results in secretion elimination and improved lung function, thus improving the breathing system. OMS improves a newborn's sucking and swallowing reflexes, which are required for successful feeding [14]. The use of OMS and chest physiotherapy in this specific patient with HIE has proved the significance of these interventions in enhancing neonatal outcomes. His general health was enhanced by attending to his secretions and food issues during therapy, which reduced the likelihood of complications developing later [15].

Many approaches are used in chest physiotherapy with the goal of increasing lung function and removing respiratory secretions. Baby HIE patients can benefit from CPT in a number of ways, including better general respiratory conditions, decreased aspiration risks, and management of high secretion levels. Other popular techniques include circulation, shaking, and light tapping [16]. CPT sessions were linked to a decrease in secretion volume clearance and an increase in oxygen saturation in infants who met the HIE criteria. The writers underscored the significance of prompt action with the aim of averting additional consequences stemming from respiratory distress [17,18].

Although an infant's preparation for oral feeding is regarded as complete when sucking, swallowing, and respiration are coordinated, no clear definition of coordination exists. It has been discovered that each of these functions has several components that mature at different times and paces. As a result, it appears that the proper functioning of sucking, swallow processing, and respiration needs to occur at two levels: first, the elements within each function must reach an appropriate functional maturation that can work in synchrony with each other to generate a proper suck, swallow process, and respiration; and second, the elements of all these distinct functions must be able to do the same at an integrative level to ensure the safety [19].

OMS refers to these exercises and sensory inputs to the mouth and pharynx muscles to enhance oromotor function and coordination. Since the two skills that are most frequently impacted are sucking and swallowing, the algorithm has been developed to support neonates with HIE in strengthening these capacities. The methods include oral massage, NNS, and tongue and lip stimulation [20,21]. OMS improved oral motor rehabilitation and led to better eating outcomes than the control group. According to the investigation, infants receiving continuous promotor therapy were less reliant on tube feedings and were far less efficient feeders [22].

An effective illustration would be a thorough care protocol that was tested on these infants and included the administration of physiotherapy. They discovered that, in comparison to infants receiving standard physiotherapy care, the infants receiving high doses (chest physiotherapy and OMS co-intervention) had noticeably greater weight gain trajectories and a significant decrease in aspiration pneumonia occurrences. 

## Conclusions

Physiotherapy and rehabilitation are critical for the treatment of infants with HIE. The combination of percussion and vibrations gives a strategy for managing and maintaining respiratory difficulties. OMS is applied to the perioral and intraoral muscles to strengthen them, allowing for normal physiologic feeding habits. It promotes a sense of calm, allowing for more attention and concentration, as some oral motor tasks enhance breathing, sucking, and feeding. Implementing these therapies early and consistently can improve immediate clinical outcomes and also promote better long-term neurodevelopmental health. Ongoing research and advancements in clinical practices are crucial to refining these care strategies and enhancing the quality of life for these at-risk infants.
